# Increased sensitivity of p53-deficient cells to anticancer agents due to loss of Pms2

**DOI:** 10.1038/sj.bjc.6600599

**Published:** 2002-10-21

**Authors:** A Fedier, U B Ruefenacht, V A Schwarz, U Haller, D Fink

**Affiliations:** Department of Obstetrics and Gynaecology, Division of Gynaecology, University Hospital of Zurich, CH-8091, Switzerland

**Keywords:** drug sensitivity, p53, DNA mismatch repair, PMS2

## Abstract

A large fraction of human tumours carries mutations in the *p53* gene. p53 plays a central role in controlling cell cycle checkpoint regulation, DNA repair, transcription, and apoptosis upon genotoxic stress. Lack of p53 function impairs these cellular processes, and this may be the basis of resistance to chemotherapeutic regimens. By virtue of the involvement of DNA mismatch repair in modulating cytotoxic pathways in response to DNA damaging agents, we investigated the effects of loss of Pms2 on the sensitivity to a panel of widely used anticancer agents in E1A/Ha-Ras-transformed *p53*-null mouse fibroblasts either proficient or deficient in Pms2. We report that lack of the *Pms2* gene is associated with an increased sensitivity, ranging from 2–6-fold, to some types of anticancer agents including the topoisomerase II poisons doxorubicin, etoposide and mitoxantrone, the platinum compounds cisplatin and oxaliplatin, the taxanes docetaxel and paclitaxel, and the antimetabolite gemcitabine. In contrast, no change in sensitivity was found after treatment with 5-fluorouracil. Cell cycle analysis revealed that both, Pms2-deficient and -proficient cells, retain the ability to arrest at the G_2_/M upon cisplatin treatment. The data indicate that the concomitant loss of Pms2 function chemosensitises p53-deficient cells to some types of anticancer agents, that Pms2 positively modulates cell survival by mechanisms independent of p53, and that increased cytotoxicity is paralleled by increased apoptosis. Tumour-targeted functional inhibition of Pms2 may be a valuable strategy for increasing the efficacy of anticancer agents in the treatment of *p53*-mutant cancers.

*British Journal of Cancer* (2002) **87**, 1027–1033. doi:10.1038/sj.bjc.6600599 www.bjcancer.com

© 2002 Cancer Research UK

## 

The tumour suppressor gene *p53* plays a central role in controlling cell cycle checkpoint regulation, DNA repair, transcription, and apoptosis upon genotoxic stress. *p53* is one of the most frequently mutated genes in human cancers and plays a critical role in the regulation of cell cycle and apoptosis (Levine, 1997). A number of studies have suggested that loss of p53 function may be a major reason underlying failure to respond to chemotherapy (Ferreira *et al*, 1999). Evidence for this notion is emerging from studies of established human cancer cell lines and from knockout mice models.

DNA mismatch repair (MMR) proteins play an important role in the maintenance of genomic stability since it corrects replicative mismatches that escape DNA polymerase proofreading. Loss of MMR results in genomic instability characterised by small insertion and deletion mutations in repetitive sequences throughout the genome. As well as being involved in carcinogenesis, loss of the MMR activity is of concern with respect to the use of chemotherapeutic agents to treat established tumours. Loss of MMR has been reported to cause resistance to some types of anticancer agents including cisplatin (Fink *et al*, 1996) and the topoisomerase II poisons (Fedier *et al*, 2001). Interestingly, loss of MLH1 function has recently been reported to result in an increased sensitivity to cisplatin in p53-mutated human colorectal adenocarcinoma cells (Vikhanskaya *et al*, 1999; Lin *et al*, 2001). However, the cell lines used in these previous studies were not truly isogenic, since in order to restore MMR activity, a whole copy of chromosome 3 carrying a wild-type copy of MLH1 was inserted into the HCT116 cells. Thus, it is conceivable that the differences in cisplatin sensitivity observed in these cell lines could have been due to one of the many genes on the inserted chromosome other than the wild-type copy of the missing MMR gene. So far no information is available on the effect of loss of *PMS2*, another MMR gene, to chemotherapeutic agents in p53-deficient cells.

Using transformed primary fibroblasts established from E1A/Ha-Ras-transfected knockout mice, we report here that p53-deficient cells are sensitised to some anticancer agents by the additional loss of the *Pms2* gene, indicating that Pms2 positively regulates cell survival by a p53-independent mechanism. Tumour-targeted functional inhibition of Pms2 may thus be an adjunct to anticancer agents in the treatment of p53*-*mutant cancers.

## MATERIALS AND METHODS

### Cell lines

The *Pms2*^−/−^*/p53*^−/−^ and *Pms2*^+*/*+^*/p53*^−/−^ cell lines, established from E1A/Ha-Ras-transformed knockout mice primary fibroblasts, were generously provided by Dr PM Glazer (Zeng *et al*, 2000). These primary fibroblasts have been produced by breeding mice heterozygous for *Pms2* to either wild-type or *p53*-null mice, and then by intercrossing progeny animals to generate mice either wild-type or null for *Pms2* in a *p53*-nullizygous background. The cells were maintained in DMEM medium supplemented with 2 mM L-glutamine (Life Technologies, Basel, Switzerland), 10% heat inactivated foetal calf serum (Oxoid, Basel, Switzerland) and penicillin/streptomycin (100 U ml^−1^/100 μg ml^−1^, Life Technologies) at 37°C in a humidified atmosphere containing 5% carbon dioxide. The *Pms2*^−/−^*/p53*^−/−^ cells as well as the *Pms2*^+*/*+^*/p53*^−/−^ cells form defined individual colonies when seeded sparsely on standard culture dishes. All the cell lines tested negative for contamination with *Mycoplasma* spp.

### Reagents

The following drugs were generous gifts: docetaxel (Aventis, Zurich, Switzerland), cisplatin, etoposide and paclitaxel (Bristol-Myers Squipp, Baar, Switzerland), mitoxantrone (Lederle, Zug, Switzerland), doxorubicin (Pharmacia & Upjohn, Dubendorf, Switzerland), 5-fluorouracil (Roche, Reinach, Switzerland), gemcitabine (Eli Lilly, Vernier, Switzerland), and oxaliplatin (Sanofi-Synthelabo, Meyrin, Switzerland).

### Antiproliferative and cytotoxicity assays

Cisplatin, oxaliplatin, doxorubicin, mitoxantrone and 5-fluorouracil were dissolved immediately before use in 0.9% NaCl solution. Etoposide and paclitaxel were prepared in DMSO, whereas docetaxel was dissolved in methanol. The final concentration of DMSO or methanol in the cultures was <0.1% at all drug concentrations and in controls. Previous experiments (data not shown) have shown that neither 0.1% DMSO nor 0.1% methanol affects the viability or growth of these cell lines.

The antiproliferative effect in response to drug treatment was determined by the colorimetric MTT-assay (Mosmann, 1983). Briefly, cells growing in the log phase were harvested by brief trypsinisation and washed once with medium containing 10% foetal calf serum. Using 96 well plates, 1000 cells were plated 24 h prior to incubation with or without the drug for 72 h at 37°C in a humidified atmosphere containing 5% carbon dioxide. A volume of 20 μl MTT in PBS to a final concentration of 0.5 mg ml^−1^ was added, followed by incubation at 37°C for 4 h, aspiration of the medium, and addition of 200 μl DMSO. Optical density was measured by the E_max_ microplate reader E9336 (Molecular Devices, Clearwater, MN, USA) at 540 nm setting the value of the cell lines in medium to 1.0 (control) and the value of the no cells blank to zero. Differences in drug sensitivity of the respective cell lines were determined from at least four independent experiments with continuous drug exposure and are reported as the concentration required to suppress proliferation by 50% (IC_50_).

In addition, one member of each drug class was tested for cytotoxic effects by means of colony forming assay and trypan blue exclusion assay. Clonogenic survival in response to drug treatment was performed by plating 1000 cells in 60 mm cell culture dishes. After 24 h, the drug was added, followed by incubation for 6 days at 37°C in a humidified atmosphere containing 5% carbon dioxide. Cells were fixed with 25% acetic acid in ethanol and stained with Giemsa. Colonies of at least 50 cells were scored visually. Each experiment was performed a minimum of four times using triplicate cultures for each drug concentration. The logarithm of relative colony formation was plotted against the concentration of the drug. The IC_50_ was estimated by linear interpolation of the logarithmic transformed relative plating efficiencies. For trypan blue exclusion assay, cells were grown to 60% confluence and incubated with or without the drug for 24 h, 48 h or 72 h. At the time points indicated, floating and adherent cells were collected. Cells were then incubated with trypan blue solution at 0.1% final concentration for 1 min, and the number of trypan blue-positive and -negative cells was determined using a haematocytometer.

### TUNEL apoptosis assay

Cells were grown to 70% confluence in 60 mm dishes in triplicate cultures and then treated with 5 μM cisplatin. Adherent and floating cells were collected at the time points indicated and washed in PBS. Cells were then fixed by dropwise addition of ice-cold 70% ethanol. Samples were stored at 4°C until further use. Samples were washed twice with PBS and resuspended in the TUNEL reaction mixture and incubated at 37°C for 2 h, according to the manufacturer's protocol (Roche Molecular Biochemicals, Basel, Switzerland). Cells were analysed by flow cytometry (EPICS ELITE, Beckmann-Coulter, Hialeah, FL, USA).

### Cell cycle analysis

Cells were grown to 70% confluence in 60 mm dishes in triplicate cultures and then incubated with or without cisplatin (1.0 μM). This drug concentration induces about a 70% growth inhibition of Pms2-deficient cells in the MTT-assay and about a 95% reduction of clonogenic survival. Cells were then harvested by brief trypsinisation and washed once in ice-cold PBS. The pellet was resuspended in 200 μl PBS and cells were fixed by dropwise addition of 4 ml ice-cold 70% ethanol and then stored at 4°C until use. After removing ethanol by centrifugation at 3000×*g* and washing twice in PBS, cells were stained in 1 ml of propidium iodide staining solution (50 μg ml^−1^ propidium iodide and 100 U ml^−1^ RNAse A in PBS) by incubation at room temperature for 60 min in the dark and then washed once in PBS containing 0.2% BSA. Samples were analysed for their DNA content by flow cytometry (EPICS ELITE, Beckmann-Coulter, Hialeah, FL, USA), and the percentage of cells in each phase was determined using the MultiCycle for Windows Software (Phoenix Flow Systems, San Diego, CA, USA).

### Statistical analysis

Mean±s.d. values were calculated for all data sets. The two-sided paired *t* test was used to compare the effects of loss of Pms2 function on drug sensitivity. *P*<0.05 was considered to be a statistically significant difference.

## RESULTS

### Growth inhibition of Pms2^+/+^/p53^−/−^ and Pms2^−/−^/p53^−/−^ cells after treatment with anticancer agents

We addressed the question as to whether the concomitant loss of Pms2 function in p53-deficient cells is associated with changes in sensitivity to a panel of clinically relevant anticancer agents. The presence or absence of p53 and Pms2 in the respective cell lines was verified by immunoblot analysis.

**Figure 1:**
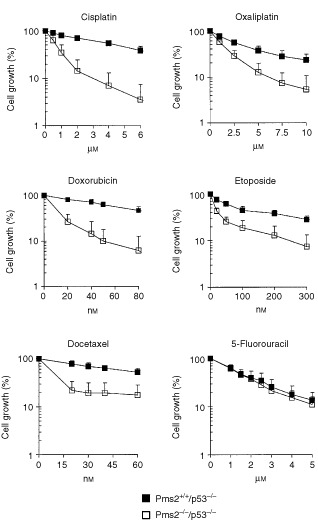
Antiproliferative effect to a continuous exposure to cisplatin, oxaliplatin, doxorubicin, etoposide, docetaxel, and 5-fluorouracil for *Pms2*^+*/*+^*/p53*^−/−^ and *Pms2*^−/−^*/p53*^−/−^ cells as determined by the MTT-assay. Each point represents the mean±s.d. of at least four independent experiments.

The antiproliferative effect of the platinum compounds cisplatin and oxaliplatin, the topoisomerase II poisons doxorubicin, mitoxantrone and etoposide, the taxanes docetaxel and paclitaxel, and the antimetabolites 5-fluorouracil and gemcitabine was tested in cell lines proficient or deficient in Pms2 in a setting of p53 nullizygosity. Figure 1

shows the antiproliferative effect to a continuous exposure to cisplatin, oxaliplatin, doxorubicin, etoposide, docetaxel and 5-fluorouracil for *Pms2*^+*/*+^*/p53*^−/−^ and *Pms2*^−/−^*/p53*^−/−^cells as determined by the MTT-assay. The Pms2-deficient *Pms2*^−/−^*/p53*^−/−^cells were 6.4-fold more sensitive to cisplatin (0.7±0.3 μM *vs* 4.7±0.6 μM, *P*=0.0002), 2.5-fold more sensitive to oxaliplatin (1.4±0.2 μM *vs* 3.5±1.1 μM, *P*=0.03), 5.3-fold more sensitive to doxorubicin (13.9±2.2 nM *vs* 73.4±18.8 nM, *P*=0.002), 3.0-fold more sensitive to mitoxantrone (2.7±0.8 nM *vs* 8.1±1.8 nM, *P*=0.005), 6.1-fold more sensitive to etoposide (17±3 nM *vs* 105±30 nM, *P*=0.01), 4.8-fold more sensitive to docetaxel (13±2 nM *vs* 64±13 nM, *P*=0.0005), 4.3-fold more sensitive to paclitaxel (15±4 nM *vs* 66±14 nM, *P*=0.004), and 5.5-fold more sensitive to gemcitabine (39±21 nM *vs* 213±65 nM, *P*=0.001) as compared to the *Pms2*^+*/*+^*/p53*^−/−^ cells. In contrast, no difference in sensitivity was found for 5-fluorouracil (1.5±0.2 μM *vs* 1.7±0.6 μM, *P*=0.43). Thus, our data indicate that the concomitant loss of *Pms2* in *p53*-deficient cells results in a hypersensitivity to platinum compounds, topoisomerase II poisons, taxanes, and gemcitabine.

### Clonogenic cell survival and trypan blue exclusion assays

**Figure 2:**
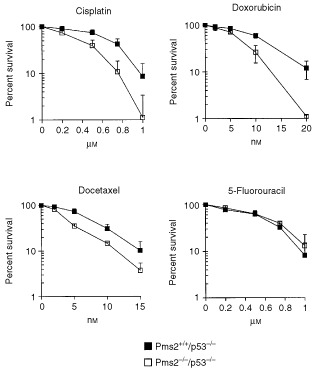
Clonogenic survival curves in response to a continuous exposure to cisplatin, doxorubicin, docetaxel, and 5-fluorouracil for the *Pms2*^+*/*+^*/p53*^−/−^ and the *Pms2*^−/−^*/p53*^−/−^ cell lines. Each point represents the mean±s.d. of at least four independent experiments.

Colorimetry-based short-term cytotoxicity assays such as the MTT-assay may underestimate overall cell killing owing to the notion that cells may not die immediately after treatment but may instead remain in a transient state of arrest for several days prior to dying (Brown and Wouters, 1999). Therefore, the clonogenic assay was performed for one member of each drug class in addition to the MTT-assay. Figure 2

shows the survival curves for the Pms2-proficient *Pms2*^+*/*+^*/p53*^−/−^ cell line as well as the repair-deficient *Pms2*^−/−^*/p53*^−/−^ cell line as a function of drug concentration. In comparison to the *Pms2*^+*/*+^*/p53*^−/−^ cell line the Pms2-deficient *Pms2*^−/−^*/p53*^−/−^ cell line was 1.6-fold more sensitive to cisplatin (0.68±0.10 μM *vs* 0.41±0.07 μM, *P*=0.002), 1.7-fold more sensitive to doxorubicin (12.1±1.4 nM *vs* 7.3±0.4 nM, *P*=0.004) and 1.8-fold more sensitive to docetaxel (7.5±1.0 nM *vs* 4.0±0.2 nM, *P*=0.005). In contrast, no difference in sensitivity was observed in response to 5-fluorouracil (0.57±0.06 μM *vs* 0.63±0.07 μM, *P*=0.25). Thus, the clonogenic survival data support the results observed in the MTT-assay, indicating that loss of Pms2 function in p53-deficient cells decreases clonogenic survival to these agents.

**Figure 3:**
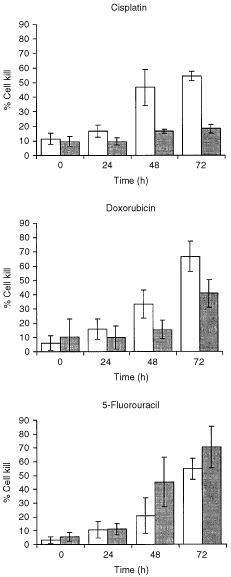
Sensitivity to cell kill of *Pms2*^−/−^*/p53*^−/−^ (open field) and *Pms2*^+*/*+^*/p53*^−/−^ (closed field) cells in response to cisplatin (3 μM), doxorubicin (50 nM) or 5-fluorouracil (5 μM) as a function of time determined by trypan blue exclusion. Each point represents the mean±s.d. of at least four independent experiments.

The trypan blue exclusion assay demonstrates that Pms2-deficient cells have a substantially higher percentage of trypan blue-stained cells than Pms2-proficient cells in response to equi-antiproliferative doses (i.e. doses that inhibited proliferation by 90% in Pms2-deficient cells) of doxorubicin (50 nM) or cisplatin (3 μM) at each time point after treatment (Figure 3

). This effect, however, is not observed in response to 5-fluorouracil (5 μM). These data indicate that Pms2 deficiency reduces the threshold for cell kill to doxorubicin and to cisplatin, but not to 5-fluorouracil. Thus, the higher sensitivity to cell kill of Pms2-deficient cells to doxorubicin or cisplatin confirms the increased antiproliferative effect and the decreased clonogenic survival of these cells to these agents.

### Pms2-deficiency and cisplatin-induced apoptosis

Using cisplatin as a representative of compounds that display hypersensitivity in clonogenic survival as well as in proliferation inhibition, the TUNEL apoptosis assay was performed to determine whether the increased sensitivity to cisplatin due to loss of Pms2 function was accompanied by increased apoptosis. Treatment with 5 μM cisplatin, which inhibited growth of Pms2-deficient cells by more than 95%, produced more apoptotic *Pms2*^−/−^*/*p53^−/−^ cells than *Pms2*^+*/*+^*/p53*^−/−^cells. The respective values were 37±5% *vs* 5±1% at 30 h after cisplatin treatment and 43±4% *vs* 20±14% at 60 h after treatment. These results indicate that the hypersensitivity to the cytotoxic effect of cisplatin in Pms2-deficient cells is associated with increased apoptosis.

### Cell cycle analysis of* Pms2^+/+^*/*p53^−/−^* and*Pms2^−/−^*/*p53^−/−^* cells after treatment with cisplatin

**Figure 4:**
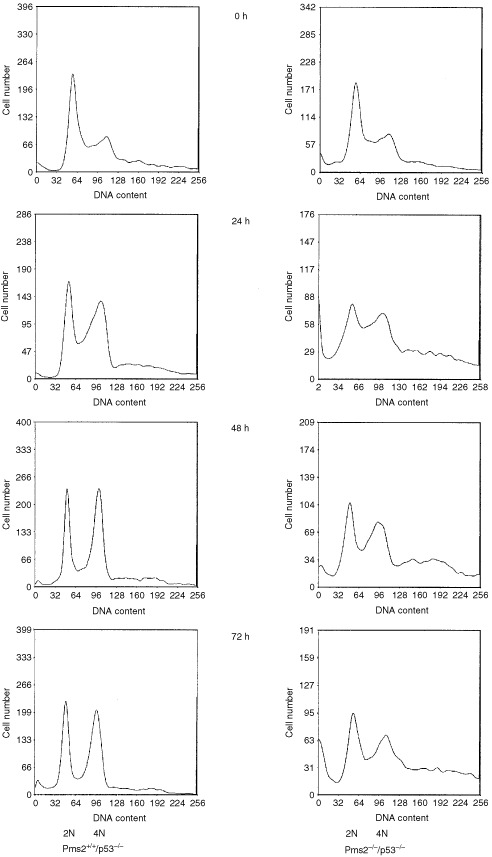
Representative cell cycle phase distribution profile of the DNA content for *Pms2*^+*/*+^*/p53*^−/−^ cells (left panel) and *Pms2*^−/−^*/p53*^−/−^ cells (right panel) as a function of time (0 h, 24 h, 48 h, 72 h) in response to treatment with 1.0 μM cisplatin. 2N represent cells accumulated in G_1_, 4N represent cells accumulated in G_2_/M.

The question was addressed as to whether the increased sensitivity to, for instance, cisplatin in p53-deficient cells is accompanied by alterations in triggering cell cycle checkpoint activation. Cisplatin has been shown to be a potent inducer of MMR-dependent G_2_/M arrest (Brown *et al*, 1997). Figure 4

shows that 1 μM cisplatin, which inhibited growth of Pms2-deficient cells by 70% in the MTT-assay, resulted in response to cisplatin in a sustained accumulation at the G_2_/M transition of Pms2-proficient and -deficient cells. The data demonstrate that both Pms2-proficient and -deficient cells retain the ability to arrest at the G_2_/M upon cisplatin treatment. Thus, the observed hypersensitivity in Pms2-deficient cells seems not to be accompanied by alterations in the characteristic changes in cell cycle distribution profile induced by drug treatment.

## DISCUSSION

The present study indicates that *p53*-null mouse fibroblasts are rendered hypersensitive to cell killing in response to platinum compounds, topoisomerase II poisons, taxanes and the antimetabolite gemcitabine by the concomitant loss of Pms2 function, and that both Pms2-deficient and -proficient cells retain the ability to arrest at G_2_/M upon cisplatin treatment. This observation is important for several reasons: First, it identifies PMS2 as another putative protective mediator of cell survival in p53-deficient cells, and it thus expands on the previous finding reporting increased sensitivity to cisplatin by the concomitant loss of MLH1 function (Vikhanskaya *et al*, 1999; Lin *et al*, 2001). Second, it identifies, in addition to the platinum compounds, two other clinically important classes of anticancer agents that result in hypersensitivity in p53-deficient cells by the additional loss of MMR. Third, it further supports the notion that MMR proteins such as PMS2 or MLH1 (Vikhanskaya *et al*, 1999; Lin *et al*, 2001) modulate protective DNA damage response pathways independently of p53 function. This finding supports the concept of a novel role of MMR, in addition to mediating drug-induced cytotoxicity (Fink *et al*, 1998; Fedier *et al*, 2001), as a positive modulator of cell survival after DNA damage. A precedent for this hypothesis has already been found as it has been reported that MLH1-deficiency is associated with hypersensitivity to mitomycin C in p53-proficient cells (Fiumicino *et al*, 2000). Fourth, the observed hypersensitivity of Pms2-deficient cells seems to be independent of the cell cycle and seems to be due to increased apoptosis. The observed p53-independence of cytotoxicity goes along with the previous observation that MMR can trigger apoptosis in a p53-independent pathway (Zeng *et al*, 2000).

Mutations that disable p53 are frequently found in human cancers (Hollstein *et al*, 1991), often in association with tumour progression or high grade malignancy (Carder *et al*, 1993). Therefore, much effort has gone into determining the effects of p53 inactivation on the response of cancer cells to therapeutic agents. The results vary with the cell line under study and the experimental set-up, and thus both decreased and increased sensitivities have been observed in different model systems (Blandino *et al*, 1999; Bunz *et al*, 1999). Recently, it has been reported that loss of MLH1 is associated with a hypersensitivity to cisplatin in p53-deficient cells in the HCT116 model (Vikhanskaya *et al*, 1999; Lin *et al*, 2001). However, the repair-deficient member of these cell lines was not truly isogenic since in order to restore MMR activity a whole copy of chromosome 3 carrying a wild-type copy of MLH1 was inserted into the HCT116 cells (Koi *et al*, 1994). The present study uses isogenic primary fibroblasts established from E1A/Ha-Ras-transfected knockout mice. These cells are genetically well defined and therefore constitute a more meaningful test system than cancer-derived cell lines, not only because they are truly isogenic but also because the cancer-derived cells are likely to contain a number of other accumulated mutations and abnormalities that could influence response to anticancer agents. It is unlikely that the hypersensitivity seen in the *Pms2*^−/−^*/p53*^−/−^ cells was due to the experimental set-up rather than being caused by the lack of Pms2 function, because the increased sensitivity was also observed, though to a lesser extent, in the clonogenic survival assay and in the trypan blue exclusion assay. This observation indicates that the increased sensitivity to the platinum compounds, the topoisomerase II poisons and the taxanes arises through enhanced cell killing rather than only through decreased proliferation ability. The increased cell killing by cisplatin in cells lacking Pms2 is paralleled by increased apoptosis.

The mechanism by which the MMR proteins may influence damage response is not yet fully understood. One hypothesis proposes that the MMR proteins recognise base damage and initiate a cycle of futile repair, leading to gaps and breaks that may ultimately signal apoptosis (Vaisman *et al*, 1998). It is also possible that the recognition of damage by the MMR proteins directly initiate a signal transduction pathway (Fink *et al*, 1998). Direct evidence supporting the latter hypothesis includes a requirement for MLH1 function in cisplatin induction of c-abl kinase activity (Nehmé *et al*, 1997) and of p73 accumulation (Gong *et al*, 1999).

An increased cytotoxic effect due to loss of Pms2 was observed in p53-deficient cells to a panel of drugs that have different modes of action and introducing different types of damage. The molecular basis for the putative protective role of MMR in the p53-independent response to platinum compounds, topoisomerase II poisons and to taxanes is not yet clear. Loss of either Pms2 or MLH1 (Vikhanskaya *et al*, 1999; Lin *et al*, 2001) results in p53-deficient cells in hypersensitivity to cisplatin, whereas loss of MLH1 or PMS2 results in p53-proficent cells in resistance to cisplatin (Aebi *et al*, 1996; Fink *et al*, 1997). Likewise, cell kill by topoisomerase II poisons, but not by taxanes, has been reported to be affected by loss of MMR (Fedier *et al*, 2001; Lin *et al*, 2001), indicating that MMR-dependent damage response is, at least partially, modulated by p53. Indeed, cross talks between MMR- and p53-dependent pathways have been reported (Duckett *et al*, 1999; Wu *et al*, 1999). Cells defective in p53 function retain the ability to mediate apoptosis by a p73-dependent pathway (Gong *et al*, 1999), which may be modulated by certain MMR proteins. It is possible that PMS2 and MLH1 protect cells from excessive cell death by counteracting p73-mediated apoptosis in a MMR-dependent manner upon DNA damage introduced by cisplatin or topoisomerase II poisons. In a p53-independent pathway PMS2 may, as in the case of cisplatin, decrease adduct tolerance and damage accumulation on this basis. Oxaliplatin and cisplatin produce different types of DNA damage and thus have been shown to display a differential response in MMR-deficient cells proficient for p53 (Fink *et al*, 1996). Our data thus suggest a role for PMS2 in a DNA damage response pathway in addition to that in MMR. However, the possibility can not be excluded that p53 modulates oxaliplatin damage differently from that of cisplatin damage. Likewise, PMS2 may modulate the efficacy of the repair machinery to process stalled replication forks arisen by blocked DNA-topoisomerase intermediates and gaps introduced by the chain-terminating antimetabolite gemcitabine. Additional loss of PMS2 thus abolishes the damage removal activity, giving rise to excessive DNA damage and cytotoxicity. The finding that MMR-deficiency does not alter 5-fluorouracil cytotoxicity in a p53-deficient setting may be of clinical interest for the treatment of colorectal cancer, despite the previously reported *in vitro* association of MMR with resistance to 5-fluorouracil in p53-proficient cells (Carethers *et al*, 1999).

p53 either mediates growth arrest, both in G_1_ or G_2_ phases of the cell cycle, or directs cells to apoptosis. These two cellular decisions are distinctive end points of p53 induction, depending on the cellular context and the type of DNA damage. The present study demonstrates that both Pms2-proficient and -deficient cells retain the ability to arrest at the G_2_/M upon cisplatin treatment and that loss of Pms2 is not accompanied by substantial alterations in the characteristic changes in cell cycle distribution. A similar result has also been reported in p53-deficient cells after loss of Msh2 (Strathdee *et al*, 2001) or MLH1 (Lin *et al*, 2001). Therefore, it seems unlikely that MMR is a major trigger for a G_2_/M arrest in cells that lack functional p53.

The feature that p53 and MMR proteins modulate cellular responses upon DNA damage and the availability of genetically engineered human and murine cells present a potential means to develop strategies to circumvent the reduced responsiveness by re-sensitising or by hypersensitising p53-mutant tumours to therapeutic regimens. Candidate approaches include the restoration of the wild-type p53 function, the activation of cytotoxic pathways that operate independently of p53, or the concurrent disruption of genes implicated in DNA damage response pathways (Mcgill and Fisher, 1999).

Since both, MMR and p53 status affect the mechanism of cytotoxicity, the genotype-based predictions may require that the MMR status as well as the p53 status of the tumour are taken into account. In summary, the present data show that p53-deficient cells are sensitised to the platinum compounds, the topoisomerase II poisons and the taxanes by the concurrent loss of Pms2 function. Although PMS2 mutations or mutations in both PMS2 and p53 are not frequently found in human cancers and thus may be of minor clinical importance, our study may nevertheless contribute to fostering the concept that tumour-targeted functional inhibition of PMS2 may be an adjunct to chemotherapy in the treatment of tumours unresponsive to therapeutic regimens due to mutations in the *p53* gene.
